# The Topp-Leone Generalized Inverted Exponential Distribution with Real Data Applications

**DOI:** 10.3390/e22101144

**Published:** 2020-10-11

**Authors:** Zakeia A. Al-Saiary, Rana A. Bakoban

**Affiliations:** Department of Statistics, College of Science, University of Jeddah, Jeddah 22254, Saudi Arabia; rabakoban@uj.edu.sa

**Keywords:** Topp-Leone distribution, generalized inverted exponential, Rényi entropy, maximum likelihood estimator, fisher information matrix, Monte Carlo simulation, 62E10, 62Q05

## Abstract

In this article, a new three parameters lifetime model called the Topp-Leone Generalized Inverted Exponential (TLGIE) Distribution is introduced. Various properties of the model are derived, including moments, quantile function, survival function, hazard rate function, mean deviation and mode. The method of maximum likelihood is used to estimate the unknown parameters. The properties of the maximum likelihood estimators using Fisher information matrix are studied. Three real data sets are applied for illustrative purpose of this study.

## 1. Introduction

Lifetime models have received great attention from statisticians, especially in the field of statistical inference. These models are of great importance in applications in many fields such as medicine, engineering, biological science, management, and public health. The Generalized Inverted Exponential (GIE) Distribution is one of these models as it is flexible to contain different forms of hazard function. It was proposed first by [1].

In recent years, researchers have proposed new families of distributions in the statistical literature by using different transformation techniques. A common technique is to introduce one or several additional tuning parameters to a standard probability distribution, with the aim to improve it, in the theoretical and practical sense. These distribution functions are more flexible to model real data, for example, the gamma-generated distribution by [2], Kumaraswamy-generated distribution by [3], McDonald-generated distribution by [4], and Weibull-generated distribution by [5], the Kumaraswamy-G family by [6] and the odd power Cauchy family by [7]. In 1955, [8] proposed a new continuous distribution that is attractive as a generator. It is known as: Topp-Leone distribution (TL). TL provides closed forms of the cumulative distribution function (cdf) and the probability distribution function (pdf). The TL distribution had not received much attention until [9] discovered it. Furthermore, there were many authors who were interested in this distribution. For example: See, [10,11,12,13,14,15,16,17,18,19,20]. In this year some authors study type II Topp-Leone, for example: see, [21,22].

So, in this paper we will introduce three parameter lifetime model called Topp-Leone Generalized Inverted Exponential Distribution. Our present study will contribute to modeling survival data. This new model was applied to three real life datasets. The first data set has to do with patients suffering from blood cancer (Leukemia) from one ministry of health hospital in Saudi Arabia. And the second data set has to do with the number of successive failures for the air conditioning system of each member in a fleet of 13 Boeing 720 jet airplanes. The third data has to do with the waiting times (in seconds), between 65 successive eruptions of the Kiama Blowhole. The results showed that the new distribution provided better fit than other distributions presented. As such, it can be categorically said that the Topp Leone Generalized Inverted Exponential distribution is good distribution in modeling survival data.

In Section 2, the pdf and cdf will be introduced. The main mathematical properties of the proposed model including, moments, survival function, hazard rate function, quantile function, mode and mean deviation will be discussed in Section 3. Moreover, Rényi entropy and fisher information will be derived in Section 4. In Section 5, we will determine the estimation of parameters. To analyze the flexibility of maximum likelihood estimators, we will provide simulation study in Section 6. Finally, three real data sets will be applied in Section 7 for illustrating purpose of this study.

The probability density function (pdf) of a two-parameter Generalized Inverted Exponential (GIE) Distribution is given by [1] as:

(1) $$g(x)=\left(\frac{\theta \lambda }{x^{2}}\right)\exp\left(-\frac{\lambda }{x}\right){\left[1-\exp\left(-\frac{\lambda }{x}\right)\right]}^{\theta -1},\;x>0,\;\lambda ,\;\theta >0,$$

and the cumulative distribution function (CDF) is given by

(2) $$G\;(x)=1-{\left[1-\exp\left(-\frac{\lambda }{x}\right)\right]}^{\theta },\;x>0,\;\lambda ,\;\theta >0,$$

where, θ is the shape parameter and λ is the scale parameter.

Recently, [15] studied Top Leone (TL) family of distributions. The cdf of TL distribution is given by:

(3) $$F_{TL-G}\;(x)\;=\;{\left[G(x)\right]}^{\alpha }\;{\left[2-G(x)\right]}^{\alpha }=\;{\left[1-{\left(\bar{G}(x)\right)}^{2}\right]}^{\alpha }\;,\;\alpha \;>\;0$$

The corresponding PDF of (3) is given by:

(4) $$f_{TL-G}\;(\;x\;)\;=\;2\;\alpha \;g\;(\;x\;)\;\bar{G}(x)\;{\left[G(x)\right]}^{\alpha -1}\;{\left[2-G(x)\right]}^{\alpha -1},\;\alpha \;>\;0$$

where $g\left(x\right)=\;\frac{dG\left(x\right)}{dx}$ considers a pdf of baseline distribution and $\bar{G}\;(x)\;=\;1\;-\;G\;(\;x\;)$. Now, we define a new lifetime model called the TLGIE distribution.

## 2. The Topp-Leone Generalized Inverted Exponential Distribution

In this section, we derive three parameter Topp-Leone generalized inverted exponential distribution. The cdf and pdf of TLGIE distribution with three parameters ( α , λ, θ ) is obtained by inserting (1) and (2) in (3) and (4):

(5) $$F\;(x)={\left[1-{\left[1-\exp\left(-\frac{\lambda }{x}\right)\right]}^{2\theta }\right]}^{\alpha },\;x>0,\;\lambda ,\;\theta ,\;\alpha \;>0,$$

and

(6) $$f\;(x)=\frac{2\theta \lambda \alpha }{x^{2}}\;\exp\;\left(-\frac{\lambda }{x}\right)\;{\left[1-\exp\left(-\frac{\lambda }{x}\right)\right]}^{2\theta -1}\;{\left[1-{\left[1-\exp\left(-\frac{\lambda }{x}\right)\right]}^{2\theta }\right]}^{\alpha -1},\;x>0,\;\lambda ,\;\theta ,\;\alpha \;>0$$

where, λ is a scale parameter and θ, α are shape parameters.

### Some Ideal Sub Models as Special Cases from Our Proposed Distribution

- For θ = 1, the proposed distribution in (5) converts to Topp-Leone Inverted Exponential (TLIE) distribution.
- For λ = 1 and θ = 1, the proposed distribution reduces to Topp-Leone Standard Inverted Exponential (TLSIE) distribution.
- For α = 1 and $\theta \;=\;\frac{1}{2}$, the proposed distribution reduces to Inverted Exponential (IE) distribution.
- For λ = 1, the proposed distribution reduces to Topp-Leone Generalized Standard Inverted Exponential (TLGSIE) distribution.
- If we replace 2θ = γ in Equation (5), we obtain: $F\;(x)={\left[1-\;{\left[1-\exp\left(-\frac{\lambda }{x}\right)\right]}^{\gamma }\right]}^{\alpha },\;x>0,\;\lambda ,\;\theta ,\;\alpha \;>0,$ the cdf of Exponentiated Generalized Inverted Exponential (EGIE) distribution with three parameters (θ, λ, α).

We can rewrite the cdf & pdf of TLGIE distribution using following series representations of [23]. 

For any real value of α,

$${\left[1+y\right]}^{\alpha }=\sum_{i\;=\;0}^{\infty }\frac{\Gamma (\alpha +1)}{j!\Gamma (\alpha +1-j)}y^{j},\alpha >0,\alpha \in R$$

The TLGIE distribution in (5) and (6) can be written as infinite sum as follows:

(7) $$\begin{array}{l} f(x)=\frac{2\theta \lambda \alpha }{x^{2}}\;\sum_{k=0}^{\infty }\sum_{j=0}^{\infty }\frac{{\left(-1\right)}^{k\;+\;j}\Gamma \;(\alpha )\;\Gamma \;(2\theta \;(1\;+\;k))\;\exp(-\frac{\lambda }{x}(1\;+\;j))}{k\;j\;\Gamma \;(k)\Gamma \;(j)\;\Gamma (\alpha \;-\;k)\;\Gamma \;(2\;\theta \;(1+k)-j)} \end{array}$$

(8) $$\begin{array}{l} f(x)=\frac{2\theta \lambda \alpha }{x^{2}}\sum_{k\;=\;0}^{\infty }\sum_{j\;=\;0}^{\infty }\frac{{\left(-1\right)}^{k\;+\;j}\exp(-\frac{\lambda }{x}(1+j))}{kj\beta (k,\alpha -k)\beta (j,2\theta (1+k)-j)} \end{array}$$

(9) $$\begin{array}{l} F(x)=\sum_{k\;=\;0}^{\infty }\sum_{j\;=\;0}^{\infty }\frac{{\left(-1\right)}^{k+j}\Gamma (\alpha +1)\Gamma (2\theta k+1)\exp(-\frac{\lambda }{x}j)}{\Gamma (k+1)\Gamma (\alpha -k+1)\Gamma (j+1)\Gamma (2\theta k-j+1)} \end{array}$$

(10) $$\begin{array}{l} F(\;x\;)=\frac{1}{\alpha \;+\;1}\;\sum_{k=0}^{\infty }\sum_{j=0}^{\infty }\frac{{\left(-1\right)}^{k\;+\;j}\exp\;(-\frac{\lambda }{x}j)}{\left(2\;\theta \;k\;+\;1)\;\beta \;(k\;+\;1,\alpha \;-\;k\;+\;1)\;\beta \;(j\;+\;1,\;2\;\theta \;k\;-\;j+1\right)} \end{array}$$

**Figure 1:**
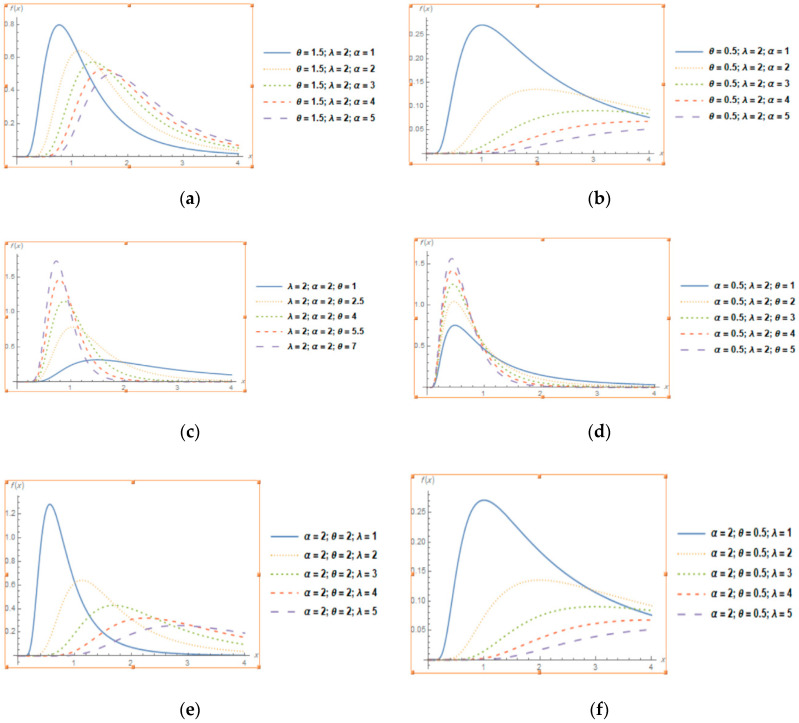
Plots of the pdf of TLGIE distribution for selected values of the parameters when (**a,b**) α increases, (**c,d**) θ increases and (**e,f**) λ increases.

Figure 1 Plots (a–f) show different shapes of the probability density functions for various values of the parameters. For these plots, it is surely clear that Topp-Leone generalized inverted exponential distribution is unimodal, right skewed and semi symmetrical distribution for some values of parameters. Therefore, according to the figures above we can assume that TLGIE distribution can be helpful in numerous applications in many fields.

## 3. Properties of TLGIE Distribution

### 3.1. Quantile and Median

The $q^{th}$ percentile of the distribution can be obtained by solving $x_{q}$ for variable X. The $q^{th}$ percentile is obtained by solving $Q\;(\;x\;)\;=\;F^{-1}\;(\;x\;)$ as:

(11) $$x_{q}\;=\;\frac{-\lambda }{\ln\;(\;1\;-\;{\left(1\;-\;q^{\frac{1}{\alpha }}\right)}^{\frac{1}{2\theta }}}$$

The Median of the TLGIE distribution can be defined at q = 0.5. We can easily generate the random sample from (11) using q as uniform random number.

### 3.2. Moments

The moments of TLGIE distribution is computed using Equation (7) as following:

(12) $$\mu_{r}{}^{\prime }=2\theta \lambda \alpha \sum_{k=0}^{\infty }\sum_{j=0}^{\infty }\frac{{\left(-1\right)}^{k+j}\Gamma \left(\alpha \right)\Gamma \left(2\theta \left(1+k\right)\right)}{kj\Gamma \left(k\right)\Gamma \left(j\right)\Gamma \left(\alpha -k\right)\Gamma (\left(2\theta \left(1+k\right)-j\right)}\;\times \int_{0}^{\infty }x^{r-2}\exp\left(-\frac{\lambda }{x}\left(1+j\right)\right)dx,$$

Making transformation as $y\;=\;\frac{\lambda }{x}\;(j\;+\;1)$ in above expression, we obtain the moments of Topp-Leone generalized inverted exponential distribution:

(13) $$\begin{array}{cc} \mu_{r}{}^{\prime } & =2\theta \lambda^{r}\alpha \sum_{k=0}^{\infty }\sum_{j=0}^{\infty }\frac{{\left(-1\right)}^{k\;+\;j}\;\Gamma \;(\alpha )\;\Gamma \;(2\;\theta \;(1\;+\;k)){\left(1\;+\;j\right)}^{r\;-\;1}}{k\;j\;\Gamma \;(k)\;\Gamma \;(j)\;\Gamma \;(\alpha \;-\;k)\;\Gamma \;(\;2\;\theta \;(1\;+\;k)-j)}\\ \; & \times \;\left({Ε}_{r}(1)+\sum_{i=0}^{\infty }\frac{{\left(-1\right)}^{i}}{(i\;-\;r\;+\;1)i!}\right) \end{array}$$

where ${Ε}_{r}(1)$ is the integration exponential function.

We can compute the coefficient of variation (CV), coefficient of skewness (CS) and coefficient of kurtosis (CK) of TLGIE distribution using (13) in the following relations:

$$\begin{array}{c} CV=\sqrt{\frac{\mu_{2}}{\mu_{1}}-1}.\\ CS=\frac{\mu_{3}-3\mu_{2}\mu_{1}+2\mu_{1}^{3}}{{\left(\mu_{2}-\mu_{1}\right)}^{\frac{3}{2}}}.\\ CK=\frac{\mu_{4}-4\mu_{3}\mu_{1}+6\mu_{2}\mu_{1}^{2}}{{\left(\mu_{2}-\mu_{1}^{2}\right)}^{2}}. \end{array}$$

CV, CS and CK are very important statistical measures for studying the behavior of the distribution.

### 3.3. Reliability Function

The TLGIE distribution is used for describing a random lifetime in reliability analysis. The reliability function of the TLGIE distribution is denoted by R ( x ), also known as survival function and obtained as follows

(14) R ( x ) = 1 − F ( x ),

The survival function of TLGIE distribution is obtained by substituting (5) in (14) to deduce:

(15) $$R\;(\;x\;)\;=\;1\;-\;{\left[1-\;{\left[1-\exp\left(-\frac{\lambda }{x}\right)\right]}^{2\;\theta }\right]}^{\alpha },$$

**Figure 2:**
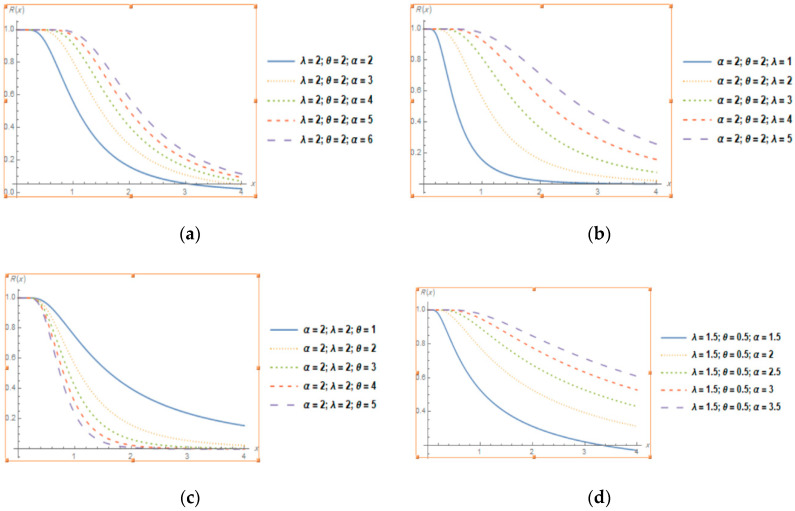
Plots of the reliability function of TLGIE distribution for selected values of the parameters when (**a,d**) α increases, (**b**) λ increases and (**c**) θ increases.

**Figure 3:**
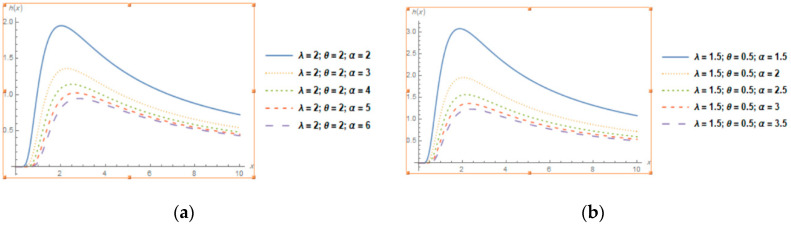

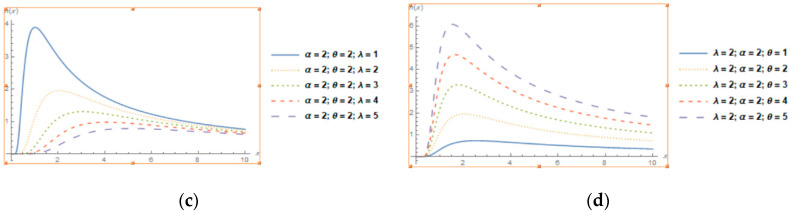
Plots of the Hazard Function of TLGIE distribution for selected values of the parameters when (**a,b**) α increases, (**c**) λ increases and (**d**) θ increases.

Figure 2 shows that the reliability curves for different values of the parameters for TLGIE distribution is decreasing. Figure 3 shows that the hazard function for different values of the parameters for TLGIE is increasing at first then decreasing in shape i.e., it takes the upside-down bathtub shaped. The lifetime models that present first increase and then decrease shaped failure rates are very useful in survival analysis. 

### 3.4. Hazard Rate Function

It is another characteristic in reliability analysis. It is denoted by h(y). For TLGIE the hazard function is defined as follows

(16) $$h\;(\;x\;)\;=\;\frac{\frac{2\;\theta \;\lambda \;\alpha }{x^{2}}\exp\;\left(-\frac{\lambda }{x}\right)\;{\left[1-\exp\;\left(-\frac{\lambda }{x}\right)\right]}^{2\;\theta \;-1}\;{\left[1\;-\;{\left[1-\exp\;\left(-\frac{\lambda }{x}\right)\right]}^{2\;\theta }\right]}^{\alpha \;-1}}{1\;-\;{\left[1-\;{\left[1-\exp\left(-\frac{\lambda }{x}\right)\right]}^{2\;\theta }\right]}^{\alpha }},$$

### 3.5. Mode

We consider the density function of TLGIE distribution given in (6) and take the first derivative with respect to x to obtain the mode of Topp-Leone generalized inverted exponential distribution as follows

(17) $$\begin{array}{c} \frac{df\left(x\right)}{dx}=f\;\left(x\right)[\frac{-2}{x}+\frac{\lambda }{x^{2}}-\left(2\theta -1\right)\frac{\lambda }{x^{2}}\exp\left(-\frac{\lambda }{x}\right){\left[1-\exp\left(-\frac{\lambda }{x}\right)\right]}^{-1}\\ +2\theta \left(\alpha -1\right)\frac{\lambda }{x^{2}}\exp\left(-\frac{\lambda }{x}\right){\left[1-\exp\left(-\frac{\lambda }{x}\right)\right]}^{2\theta -1}{\left[1-{\left[1-\exp\left(-\frac{\lambda }{x}\right)\right]}^{2\theta }\right]}^{-1}], \end{array}$$

By putting $\frac{df\left(x\right)}{dx}=0$, the maxima can be obtained by solving (17) iteratively using numerical methods as Newton- Raphson. 

The mode, median, mean, skewness and kurtosis of the TLGIE distribution for various values of α, θ and λ shown in Table 1 and Table 2.

From Table 1 and Table 2, we can study the behavior of the TLGIE distribution by changing the parameter values. We can deduce that if α increases, the mode, median and mean are increased but the skewness and kurtosis are decreased. If θ increases, the mode, median and mean are decreased, else the skewness and kurtosis are decrease. If λ increase, the mode, median and mean are decrease but the skewness and kurtosis remain the same. In any values of parameters, we observe that mode < median < mean, this means that the TLGIE distribution is always right skewed and unimodal.

### 3.6. The Mean Deviation and the Median Deviation

The mean deviation is a measure of dispersion derived by computing the mean of the absolute values of the differences between the observed values of a variable and the mean or median of the variable. Also, it is called average deviation. The mean deviation about the mean is defined by:

(18) $$\begin{array}{cc} D\;(\mu ) & =\;Ε\;\left|x\;-\;\mu \right|\\ \; & =\;\int_{0}^{\infty }\left|x-\mu \right|f\;(x)\;dx\\ \; & =\;\int_{0}^{\mu }(x\;-\;\mu )f\;(x)\;dx\;+\;\int_{\mu }^{\infty }(x\;-\;\mu )f\;(x)\;dx\\ \; & =\;2\;\mu \;F\;(\mu )\;-2\int_{0}^{\mu }x\;dF\;(x)\\ \; & =\;2\;\int_{0}^{\mu }F\;(x)\;dx, \end{array}$$

By substituting from Equation (9) in (18), we obtain the mean deviation about the mean as:

(19) $$\begin{array}{c} \begin{array}{c} D(\mu )=2\sum_{k\;=\;0}^{\infty }\sum_{j\;=\;0}^{\infty }\frac{{\left(-1\right)}^{k+j}\Gamma (\alpha +1)\Gamma (2\theta k+1)}{\Gamma (k+1)\Gamma (\alpha -k+1)\Gamma (j+1)\Gamma (2\theta k-j+1)}\times \\ \int_{0}^{\mu }\exp\left(-\frac{\lambda }{x}j\right)dx\\ =2\sum_{k\;=\;0}^{\infty }\sum_{j\;=\;0}^{\infty }\frac{{\left(\;-\;1\;\right)}^{k\;+\;j}\Gamma (\alpha +1)\Gamma (2\theta k+1)}{\Gamma (k+1)\Gamma (\alpha -k+1)\Gamma (j+1)\Gamma (2\theta k-j+1)} \end{array},\\ \times \left(\mu \exp\left(-\frac{\lambda }{\mu }j\right)-\lambda j\Gamma (0,\frac{\lambda }{\mu }j)\right),(\frac{\lambda }{\mu }j)\in R>0 \end{array}$$

where, $\Gamma (0,\frac{\lambda }{\mu }j)$ is the incomplete gamma function.

Next, the mean deviation about the median is obtained as:

(20) $$\begin{array}{cc} D\;(\;m\;) & =\;Ε\;\left|\;x\;-\;m\;\right|\\ \; & =\;\mu \;-\;m\;+\;2\;\int_{0}^{m}F\;(\;x\;)\;dx \end{array},$$

And for TLGIE, by substituting from Equation (9) in (20), we obtain the median deviation as:

(21) $$\begin{array}{c} \begin{array}{c} D(m)=\mu -m+2\sum_{k\;=\;0}^{\infty }\sum_{j\;=\;0}^{\infty }\frac{{\left(-1\right)}^{k+j}\Gamma (\alpha +1)\Gamma (2\theta k+1)}{\Gamma (k+1)\Gamma (\alpha -k+1)\Gamma (j+1)\Gamma (2\theta k-j+1)}\times \\ \int_{0}^{m}\exp\left(-\frac{\lambda }{x}j\right)dx\\ =\mu -m+2\sum_{k\;=\;0}^{\infty }\sum_{j\;=\;0}^{\infty }\frac{{\left(\;-\;1\;\right)}^{k\;+\;j}\Gamma (\alpha +1)\Gamma (2\theta k+1)}{\Gamma (k+1)\Gamma (\alpha -k+1)\Gamma (j+1)\Gamma (2\theta k-j+1)} \end{array}\\ \times \left(m\exp\left(-\frac{\lambda }{m}j\right)-\lambda j\Gamma (0,\frac{\lambda }{m}j)\right),(\frac{\lambda }{m}j)\in R>0 \end{array}$$

where, $\Gamma (0,\frac{\lambda }{m}j)$ was known in (19).

## 4. Rényi Entropy of TLGIE

In the present section, we provide an important measure, the Rényi entropy. It was introduced by [24]. It is one of the several generalizations of Shannon’s entropy, see [25]. The theory of entropy has been successfully used in a wide diversity of applications such as in information theory, engineering, and physics, see [26]. Entropy is defined in physics via the second law of thermodynamics. Thermodynamic system that is also usually considered to be a measure of the system’s disorder, that is a property of the system’s state, and that varies directly with any reversible change in heat in the system and inversely with the temperature of the system. In this paper, we interest in the statistical mechanics of entropy. The interpretation of entropy in statistical mechanics is the measure of uncertainty, which remains about a system after its observable macroscopic properties, such as temperature, pressure and volume, have been taken into account. The entropy of a probability distribution can be interpreted not only as a measure of uncertainty but also as a measure of information. It has also been used for the characterization of numerous standard probability distributions. For the density function *f* (*x*), the Rényi entropy is defined by:

(22) $$R_{\beta }\;(\;x\;)\;=\;\frac{1}{1-\beta }\;Log\;[\;J\;(\;\beta \;)\;]$$

where

(23) $$J\;(\;\beta \;)\;=\;\int_{0}^{\infty }f^{\beta }\;(\;x\;)\;dx;\;\beta \;\neq \;1$$

By substituting from Equation (9) in (23), we obtain:

(24) $$\begin{array}{cc} J(\beta ) & ={\left(2\;\alpha \;\theta \;\lambda \right)}^{\beta }\sum_{k\;=\;0}^{\infty }\sum_{j\;=\;0}^{\infty }{\left(-1\right)}^{k+j}(\begin{array}{c} \beta (\alpha -1)\\ k \end{array})(\begin{array}{c} 2\theta (\beta +k)-\beta \\ j \end{array})\int_{0}^{\infty }x^{-2\beta }\exp\left(-\frac{\lambda }{x}(k+\beta )\right)dx\\ \; & ={\left(2\;\alpha \;\theta \;\lambda \right)}^{\beta }\Gamma \left(2\beta -1\right)\sum_{k\;=\;0}^{\infty }\sum_{j\;=\;0}^{\infty }\frac{(-1{)}^{k\;+\;j}}{{\left[\lambda \;(\;k\;+\;\beta \;)\right]}^{2\;\beta \;-\;1}}\times \\ & \frac{{\left(-1\right)}^{k+j}\Gamma (\beta (\alpha -1)+1)\Gamma (2\theta (\beta +k)-\beta +1)}{\Gamma (k+1)\Gamma (j+1)\Gamma (\beta (\alpha -1)-k+1)\Gamma (2\theta (\beta +k)-\beta -j+1)} \end{array},$$

Thus, the Rényi entropy for TLGIE distribution is

$$R_{\beta }\;(\;x\;)\;=\;\frac{1}{1-\beta }\;Log\;\left[\begin{array}{c} {\left(2\;\alpha \;\theta \;\lambda \right)}^{\beta }\Gamma \left(2\beta -1\right)\sum_{k\;=\;0}^{\infty }\sum_{j\;=\;0}^{\infty }\frac{{\left(\;-\;1\;\right)}^{k\;+\;j}}{{\left[\lambda \;(\;k\;+\;\beta \;)\right]}^{2\;\beta \;-\;1}}\times \\ \frac{{\left(-1\right)}^{k+j}\Gamma (\beta (\alpha -1)+1)\Gamma (2\theta (\beta +k)-\beta +1)}{\Gamma (k+1)\Gamma (j+1)\Gamma (\beta (\alpha -1)-k+1)\Gamma (2\theta (\beta +k)-\beta -j+1)} \end{array}\right]$$

## 5. Parameters Estimation

### 5.1. Maximum Likelihood Estimation

In this section, we derive the maximum likelihood estimates (MLE_s_) and inference for unknown parameters of Topp-Leone Generalized Inverted Exponential distribution. Let $x_{1},\;x_{2},\;\ldots \;,\;x_{n}$ be a realization of a random sample of size n from TLGIE distribution then the likelihood function is written as follows

$$L=\overset{n}{\underset{i=0}{\prod }}f\left(y_{i}\right),$$

and the log-likelihood function is given as follows

(25) $$\begin{array}{cc} \ell \;=\;\log\;(\;L\;) & =\;n\;\log\;(\;2\;\alpha \;\theta \;\lambda \;)\;-\;2\;\sum_{i=1}^{n}\log\;(\;x_{i})\;-\;\sum_{i=1}^{n}\;\frac{\lambda }{x_{i}}\;+\;(\;2\;\theta \;-\;1\;)\;\sum_{i=1}^{n}\;\log\;(\;1\;-\;e^{-\;\frac{\lambda }{x_{i}}\;})\\ \; & +\;(\;\alpha \;-\;1\;)\;\sum_{i=1}^{n}\;\log\;\left(1\;-\;{\left(\;1\;-\;e^{-\;\frac{\lambda }{x_{i}}\;}\right)}^{\;2\;\theta }\right) \end{array},$$

Differentiating (25) with respect α,θ,λ, respectively, and equating them to 0, we have

(26) $$\frac{n}{\alpha }\;+\;\sum_{i=1}^{n}\;\log\;\left(1\;-\;{\left(\;1\;-\;e^{-\;\frac{\lambda }{x_{i}}\;}\right)}^{\;2\;\theta }\;\right)\;=\;0,$$

(27) $$\;\frac{n}{\theta }\;+\;2\;\sum_{i=1}^{n}\;\log\;(\;1\;-\;e^{-\;\frac{\lambda }{x_{i}}\;})\;-\;2\;(\;\alpha \;-\;1\;)\;\sum_{i=1}^{n}\frac{{\left(\;1\;-\;e^{-\;\frac{\lambda }{x_{i}}\;}\right)}^{\;2\;\theta }\log\;(\;1\;-\;e^{-\;\frac{\lambda }{x_{i}}\;})}{1\;-\;{\left(\;1\;-\;e^{-\;\frac{\lambda }{x_{i}}\;}\right)}^{\;2\;\theta }\;}\;=\;0,$$

(28) $$\;\frac{n}{\lambda }\;-\;\sum_{i=1}^{n}\;\frac{1}{x_{i}}\;+\;(\;2\;\theta \;-\;1\;)\;\sum_{i=1}^{n}\frac{x_{i}{}^{-\;1}\;e^{-\;\frac{\lambda }{x_{i}}}}{\;1\;-\;e^{-\;\frac{\lambda }{x_{i}}\;}}\;-\;2\;\theta \;(\;\alpha \;-\;1\;)\;\sum_{i=1}^{n}\;\frac{x_{i}{}^{-\;1}\;e^{-\;\frac{\lambda }{x_{i}}\;}\;{\left(\;1\;-\;e^{-\;\frac{\lambda }{x_{i}}\;}\right)}^{\;2\;\theta \;-\;1}}{1\;-\;{\left(\;1\;-\;e^{-\;\frac{\lambda }{x_{i}}\;}\right)}^{\;2\;\theta }}\;=\;0,$$

The maximum likelihood estimates of α, θ and  λ are obtained iteratively by solving (26), (27), and (28), simultaneously.

### 5.2. Fisher Information

The approximate variance covariance matrix of the (MLE_s_) for the parameters of TLGIE distribution with $\underline{\gamma }=\left(\overset{\wedge }{\alpha },\overset{\wedge }{\theta },\overset{\wedge }{\lambda }\right)$ is obtained by

$$\begin{array}{c} {\overset{\wedge }{I}}_{n}^{-1}\left(\overset{\wedge }{\underline{\gamma }}\right)=\left(\begin{array}{ccc} \mathrm{var}(\overset{\wedge }{\alpha )} & \mathrm{cov}(\overset{\wedge }{\alpha },\overset{\wedge }{\theta )} & \mathrm{cov}(\overset{\wedge }{\alpha },\overset{\wedge }{\lambda )}\\ \mathrm{cov}(\overset{\wedge }{\theta ,}\overset{\wedge }{\alpha )} & \mathrm{var}(\overset{\wedge }{\theta )} & \mathrm{cov}(\overset{\wedge }{\theta },\overset{\wedge }{\lambda )}\\ \mathrm{cov}(\overset{\wedge }{\lambda ,}\overset{\wedge }{\alpha )} & \mathrm{cov}(\overset{\wedge }{\lambda },\overset{\wedge }{\theta }) & \mathrm{var}(\overset{\wedge }{\lambda }) \end{array}\right)\\ {\overset{\wedge }{I}}_{n}^{-1}\left(\overset{\wedge }{\underline{\gamma }}\right)={\left(-\left(\frac{\partial^{2}\log L}{\partial \gamma_{i}\partial \gamma_{j}}\right)\right)}_{\gamma =\overset{\wedge }{\gamma }} \end{array}$$

The elements of the observed Fisher information matrix, could be found by using the second partial derivatives of the maximum likelihood estimators as follows

(29) $$\frac{\partial^{2}\;\log\;L}{\partial \alpha^{2}}\;=\;-\;\frac{n}{\alpha^{2}}\;,$$

(30) $$\;\frac{\partial^{2}\;\log\;L}{\partial \alpha \;\partial \theta }\;=\;-\;2\;\sum_{i=1}^{n}\;\frac{{\left(\;1\;-\;e^{-\;\frac{\lambda }{x_{i}}\;}\right)}^{2\;\theta }\log\;(\;1\;-\;e^{-\;\frac{\lambda }{x_{i}}\;})}{1\;-\;{\left(\;1\;-\;e^{-\;\frac{\lambda }{x_{i}}\;}\right)}^{2\;\theta }}\;,$$

(31) $$\frac{\partial^{2}\;\log\;L}{\partial \alpha \;\partial \lambda }\;=\;-\;2\;\theta \;\sum_{i=1}^{n}\;\frac{e^{-\;\frac{\lambda }{x_{i}}\;}{\left(\;1\;-\;e^{-\;\frac{\lambda }{x_{i}}\;}\right)}^{2\;\theta \;+1}}{x_{i}\;\left(1\;-\;{\left(\;1\;-\;e^{-\;\frac{\lambda }{x_{i}}\;}\right)}^{2\;\theta }\right)\;}\;,$$

(32) $$\frac{\partial^{2}\;\log\;L}{\partial \theta^{2}}\;=\;-\;\frac{n}{\theta^{2}}\;+\;4\;(\;1\;-\;\alpha \;)\;\sum_{i=1}^{n}\;\frac{{\left(\;1\;-\;e^{-\;\frac{\lambda }{x_{i}}\;}\right)}^{2\;\theta \;}\;\log\;{\left(\;1\;-\;e^{-\;\frac{\lambda }{x_{i}}\;}\right)}^{2\;}\;}{{\left(1\;-\;{\left(\;1\;-\;e^{-\;\frac{\lambda }{x_{i}}\;}\right)}^{2\;\theta }\right)}^{2}\;}\;,$$

(33) $$\begin{array}{cc} \frac{\partial^{2}\;\log\;L}{\partial \theta \;\partial \lambda } & =\;-\;2\;(\;1\;-\;\alpha \;)\sum_{i=1}^{n}\;\frac{e^{-\;\frac{\lambda }{x_{i}}\;}{\left(\;1\;-\;e^{-\;\frac{\lambda }{x_{i}}\;}\right)}^{2\;\theta \;-1}\left(1\;+\;2\;\theta \;\log\;(\;1\;-\;e^{-\;\frac{\lambda }{x_{i}}\;})-{\left(\;1\;-\;e^{-\;\frac{\lambda }{x_{i}}\;}\right)}^{2\;\theta }\right)\;}{x_{i}\;{\left(1\;-\;{\left(\;1\;-\;e^{-\;\frac{\lambda }{x_{i}}\;}\right)}^{2\;\theta }\right)}^{2}\;}\\ \; & +\;2\;\sum_{i=1}^{n}\;\frac{e^{-\frac{\lambda }{x_{i}}}}{x_{i}\;(\;1\;-\;e^{-\;\frac{\lambda }{x_{i}}\;})} \end{array},$$

(34) $$\frac{\partial^{2}\;\log\;L}{\partial \lambda^{2}}\;=\;-\;\frac{n}{\lambda^{2}}\;+\;(\;2\;\theta \;-\;1\;)\;\sum_{i=1}^{n}\;\frac{\;e^{-\;\frac{\lambda }{x_{i}}\;}\;}{x_{i}^{2}\;{\left(\;1\;-\;e^{-\;\frac{\lambda }{x_{i}}\;}\right)}^{2\;}\;}\;+2\;\theta \;(\;\alpha -\;1\;)\;\sum_{i=1}^{n}\;\frac{e^{-\;\frac{\lambda }{x_{i}}\;}{\left(\;1\;-\;e^{-\;\frac{\lambda }{x_{i}}\;}\right)}^{2\;\theta \;-\;1\;}\psi_{i}\;}{x_{i}^{2}\;{\left(1\;-\;{\left(\;1\;-\;e^{-\;\frac{\lambda }{x_{i}}\;}\right)}^{2\;\theta }\right)}^{2}\;}\;,$$

where: $\psi_{i}\;=\;1\;-\;{\left(\;1\;-\;e^{-\;\frac{\lambda }{x_{i}}\;}\right)}^{2\;\theta }-\;(\;2\;\theta \;-\;1\;)\;e^{-\;\frac{\lambda }{x_{i}}\;}\;{\left(\;1\;-\;e^{-\;\frac{\lambda }{x_{i}}\;}\right)}^{-1\;}\;-\;e^{-\;\frac{\lambda }{x_{i}}\;}\;{\left(\;1\;-\;e^{-\;\frac{\lambda }{x_{i}}\;}\right)}^{\;2\;\theta \;-1\;}\;$.

## 6. Simulation Study

In this section, we discuss some simulations for different sample size to determine the efficiency of MLEs. We can generate a random variable X from TLGIE using Mathematica (V.11.0). We generate samples of size n = 50; 100; 200; 500 and 1000 from TLGIE distribution for some selected combination of parameters. This process is repeated N = 1000 time to calculate mean estimate, means squared error and bias. Obtained results are given in following tables.

From Table 3, we observed that when sample size increases the mean squared error (MSE) and bias (BIAS) decrease. Therefore, the maximum likelihood method works very well to estimate the parameters of TLGIE distribution.

## 7. Applications

In this section, we provide the application with real data sets to assess the flexibility of TLGIE distribution. The parameters are estimated using maximum likelihood method.

Mathematica (V.11.0) is used for computation. We describe data sets to find the MLEs of the parameters. To assess the fitness of the real data for proposed distribution, we compared the fitness with Topp-Leone Inverted Exponential distribution (TLIE), Topp-Leone Standard Inverted Exponential distribution (TLSIE), Inverted Exponential distribution (IE) and Topp-Leone Generalized Standard Inverted Exponential distribution (TLGSIE). The required numerical evaluations are carried out using the Mathematica (V.11.0) software. In order to compare the four distribution models, we consider the criteria like AIC (Akaike information criterion), CAIC (consistent Akaike information criteria), see: [27], and HQIC (Hannan-Quinn information criterion), see: [28]. The better distribution corresponds to lesser AIC, CAIC and HQIC values.

In the following, we considered three data sets:

### 7.1. Data Set 1

The first data set that we considered, see [29], represent 40 patients suffering from blood cancer (Leukemia) from one ministry of health hospital in Saudi Arabia. The ordered life time (in years) are given as follows: 0.315, 0.496, 0.616, 1.145, 1.208, 1.263, 1.414, 2.025, 2.036, 2.162, 2.211, 2.370, 2.532, 2.693, 2.805, 2.910, 2.912, 3.192, 3.263, 3.348, 3.348, 3.427, 3.499, 3.534, 3.767, 3.751, 3.858, 3.986, 4.049, 4.244, 4.323, 4.381, 4.392, 4.397, 4.647, 4.753, 4.929, 4.973, 5.074, 4.381.

### 7.2. Data Set 2

The second data set consists of the number of successive failures for the air conditioning system of each member in a fleet of 13 Boeing 720 jet airplanes, see [30]. The actual data are: 194, 413, 90, 74, 55, 23, 97, 50, 359, 50, 130, 487, 57, 102, 15, 14, 10, 57, 320, 261, 51, 44, 9, 254, 493, 33, 18, 209, 41, 58, 60, 48, 56, 87, 11, 102, 12, 5, 14, 14, 29, 37, 186, 29, 104, 7, 4, 72, 270, 283, 7, 61, 100, 61, 502, 220, 120, 141, 22, 603, 35, 98, 54, 100, 11, 181, 65, 49, 12, 239, 14, 18, 39, 3, 12, 5, 32, 9, 438, 43, 134, 184, 20, 386, 182, 71, 80, 188, 230, 152, 5, 36, 79, 59, 33, 246, 1, 79, 3, 27, 201, 84, 27, 156, 21, 16, 88, 130, 14, 118, 44, 15, 42, 106, 46, 230, 26, 59, 153, 104, 20, 206, 5, 66, 34, 29, 26, 35, 5, 82, 31, 118, 326, 12, 54, 36, 34, 18, 25, 120, 31, 22, 18, 216, 139, 67, 310, 3, 46, 210, 57, 76, 14, 111, 97, 62, 39, 30, 7, 44, 11, 63, 23, 22, 23, 14, 18, 13, 34, 16, 18, 130, 90, 163, 208, 1, 24, 70, 16, 101, 52, 208, 95, 62, 11, 191, 14, 7.

### 7.3. Data Set 3

This data set consists of the waiting times (in seconds), between 65 successive eruptions of the Kiama Blowhole. These values were recorded with the aid of digital watch on 12 July 1998 by Jim Irish and has been referenced by [31] and [16]. The actual data are: 83, 51, 87, 60, 28, 95, 8, 27, 15, 10, 18, 16, 29, 54, 91, 8, 17, 55, 10, 35, 47, 77,36, 17, 21, 36, 18, 40, 10, 7, 34, 27, 28, 56, 8, 25, 68, 146, 89, 18, 73, 69, 9, 37, 10, 82, 29,8, 60, 61, 61, 18, 169, 25, 8, 26, 11, 83, 11, 42, 17, 14, 9, 12.

In Table 4, Table 5 and Table 6, the values of log-likelihood (LL), AIC, CAIC and HQIC are minimum and favorable of TLGIE distribution than other existing distributions, which indicates that the new model (TLGIE) is better. It is depicted from the results that our proposed model provides better than other sub models. It is be more reliable with these types of data. 

**Figure 4:**
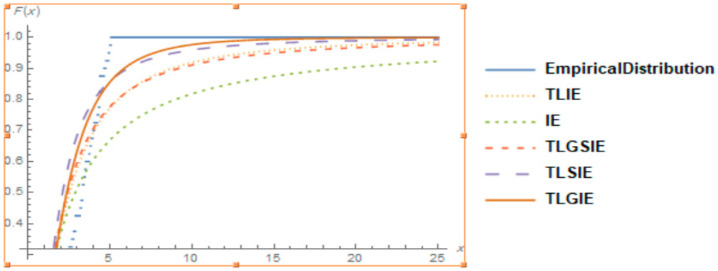
Plots of the Goodness of Fit of TLGIE distribution using data set 1.

**Figure 5:**
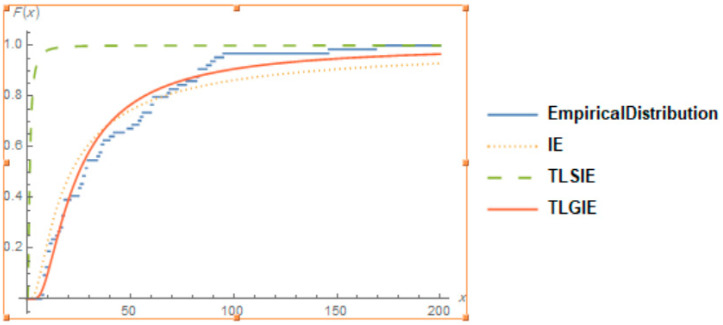
Plots of the Goodness of Fit of TLGIE distribution using data set 2.

**Figure 6:**
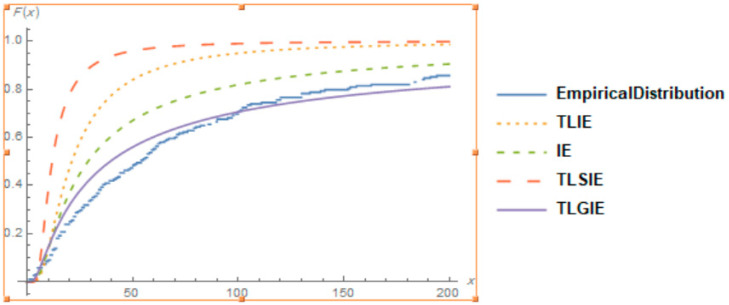
Plots of the Goodness of Fit of TLGIE distribution using data set 3.

It is also clear from Figure 4, Figure 5 and Figure 6, that the TLGIE distribution provides the best fit as compare to TLIE, TLSIE, IE and TLGSIE for given three data sets. So, the TLGIE model could be chosen as the best model.

## 8. Conclusions

We derived a three parameter Topp-Leone generalized inverted exponential distribution. Some of desirable properties are computed. The parameters are estimated by method of maximum likelihood. Performance of MLE’s are tested through simulation study. Finally, three real data applications are analyzed to assess the flexibility of new model over existing distribution. It is significantly observed that the proposed model provides better result than derived models.

## Figures and Tables

**Table 1:** The mode, median, mean, skewness and kurtosis of the TLGIE distribution for λ=2,θ={1,1.5,2} and α={1,1.5,2}.

α	Mode	Median	Mean	Skewness	Kurtosis
θ=1,λ = 2					
1	0.883857	1.62873	2.77259	0.329501	1.01815
1.5	1.21014	2.13383	3.53576	0.323435	0.998774
2	1.49385	2.56696	4.18599	0.320047	0.988074
θ=1.5,λ = 2					
1	0.813107	1.26708	1.72609	0.266825	0.783853
1.5	1.06167	1.58027	2.10295	0.260112	0.764511
2	1.26368	1.83326	2.40667	0.25654	0.754304
θ=2,λ = 2					
1	0.763937	1.08802	1.35919	0.230164	0.66022
1.5	0.96812	1.32112	1.6177	0.223727	0.642568
2	1.12748	1.50317	1.81971	0.220472	0.63372

**Table 2:** The mode, median, mean, skewness and kurtosis of the TLGIE distribution for θ=2,λ={1.5,2,2.5} and α={1,1.5,2.5}.

α	Mode	Median	Mean	Skewness	Kurtosis
θ=2,λ = 1.5					
1	0.572953	0.816016	1.01939	0.230164	0.66022
1.5	0.72609	0.990843	1.21328	0.223727	0.642568
2.5	0.944791	1.24077	1.49067	0.218523	0.628463
θ=2,λ = 2					
1	0.763937	1.08802	1.35919	0.230164	0.66022
1.5	0.96812	1.32112	1.6177	0.223727	0.642568
2.5	1.25972	1.65436	1.98757	0.218523	0.628463
θ=2,λ = 2.5					
1	0.954922	1.36003	1.69899	0.230164	0.66022
1.5	1.21015	1.65141	2.02213	2.02213	0.642568
2.5	1.57465	2.06794	2.48446	0.218523	0.628463

**Table 3:** Estimated Mean, MSE_s_ and BIAS of TLGIE distribution.

TrueValues:α=1 θ=1 λ=1				
n		$\overset{\wedge }{\alpha }$	$\overset{\wedge }{\theta }$	$\overset{\wedge }{\lambda }$
50	MLE	1.64944	1.3656	1.63393
	MSE	2.54294	0.935221	2.53382
	BIAS	0.649439	0.365595	0.633934
100	MLE	1.53764	1.2062	1.481
	MSE	2.04645	0.312122	1.76203
	BIAS	0.537636	0.2062	0.481003
200	MLE	1.48996	1.09878	1.28113
	MSE	1.72886	0.0987432	0.980768
	BIAS	0.489958	0.0987782	0.281133
500	MLE	1.28438	1.03796	1.11058
	MSE	0.888928	0.0282187	0.34375
	BIAS	0.284384	0.0379592	0.110576
1000	MLE	1.12872	1.02304	1.0703
	MSE	0.359509	0.0125671	0.168361
	BIAS	0.12872	0.0230431	0.0703043

**Table 4:** Parameters Estimation for Various Distributions depending on data set 1.

Model	Parameters			LL	AIC	CAIC	HQIC
	$\overset{\wedge }{\alpha }$	$\overset{\wedge }{\theta }$	$\overset{\wedge }{\lambda }$				
TLGIE	0.418685	2.19025	7.26267	−82.2875	170.575	171.242	172.407
TLIE	0.589171		4.55247	−85.5231	175.046	175.37	176.267
TLSIE	4.55482			−90.3942	182.788	182.894	183.399
IE			2.00825	−91.1589	184.318	184.423	184.929
TLGSIE	3.2155	0.755551		−88.1251	180.25	180.575	181.472

**Table 5:** Parameters Estimation for Various Distributions depending on data set 2.

Model	Parameters			LL	AIC	CAIC	HQIC
	$\overset{\wedge }{\alpha }$	$\overset{\wedge }{\theta }$	$\overset{\wedge }{\lambda }$				
TLGIE	8.84653	0.361313	1.11353	−1065.13	2136.25	2136.38	2140.18
TLIE	1.20401		22.9514	−1164.41	2332.83	2332.89	2335.45
TLSIE	106.161			−1379.43	2762.86	2762.92	2765.4
IE			19.9992	−1082.51	2167.01	2167.03	2168.32

**Table 6:** Parameters Estimation for Various Distributions depending on data set 3.

Model	Parameters			LL	AIC	CAIC	HQIC
	$\overset{\wedge }{\alpha }$	$\overset{\wedge }{\theta }$	$\overset{\wedge }{\lambda }$				
TLGIE	2.06861	0.77448	14.7643	−295.07	596.14	596.54	598.691
TLSIE	283.888			−304.914	611.828	611.893	612.679
IE			20.4134	−299.175	600.351	600.415	601.201

## Abbreviations

TLGIE	Topp-Leon Generalized Inverted Exponential
GIE	Generalized Inverted Exponential
TL	Topp-Leone
cdf	cumulative distribution function
pdf	probability distribution function
TLIE	Topp-Leone Inverted Exponential
TLSIE	Topp-Leone Standard Inverted Exponential
IE	Inverted Exponential
TLGSIE	Topp-Leone Generalized Standard Inverted Exponential
EGIE	Generalized Inverted Exponential
${Ε}_{r}(1)$	the integration exponential function
CV	coefficient of variation
CS	coefficient of skewness
CK	coefficient of kurtosis
MLE	maximum likelihood estimate
MSE	mean squared error
AIC	Akaike information criterion
CAIC	consistent Akaike information criterion
HQIC	Hannan-Quinn information criterion
MLE_s_	the maximum likelihood estimates
L	The likelihood function
ℓ	the log-likelihood function
BIAS	bias
LL	log-likelihood
g(x)	pdf of GIE
G(x)	Cdf of GIE
$F_{TL-G}(x)$	Cdf of TL distribution
$f_{TL-G}(x)$	Pdf of TL distribution
R (x)	The reliability or survival function
h(y)	The hazard function
D(μ)	The mean deviation
D(m)	The median deviation
